# Deformation Behaviour of Optimised Three-Dimensional Axisymmetric Chiral Auxetic Structures

**DOI:** 10.3390/biomedicines13112816

**Published:** 2025-11-18

**Authors:** Nejc Novak, Alen Grebo, Matej Borovinšek, Lovre Krstulović-Opara, Zoran Ren, Matej Vesenjak

**Affiliations:** 1 Faculty of Mechanical Engineering, University of Maribor, 2000 Maribor, Slovenia; matej.borovinsek@um.si (M.B.); zoran.ren@um.si (Z.R.); matej.vesenjak@um.si (M.V.); 2 Faculty of Electrical Engineering, Mechanical Engineering and Naval Architecture, University of Split, 21000 Split, Croatia; agrebo@fesb.hr (A.G.); opara@fesb.hr (L.K.-O.)

**Keywords:** auxetic, axisymmetric chiral structures, 3D printing, mechanical testing, deformation behaviour, optimisation

## Abstract

**Background/Objectives:** Developing functional tissue constructs via 3D bioprinting relies heavily on scaffold architecture, demanding precise mechanical tunability and high-resolution feature fidelity. **Methods:** This paper presents a novel approach utilising photocurable resins and resin 3D printing to fabricate auxetic axisymmetric chiral structures (ACSs), which can be used for advanced scaffold engineering. **Results:** The experimental tests showed that the optimised ACS (optACS) possess superior mechanical properties compared to their non-optimised counterpart. Both analysed structures possess an auxetic behaviour up to 40% longitudinal strain, with a Poisson’s ratio of about −0.1. **Conclusions:** This auxetic capability is promising for biomedical applications, particularly in developing enhanced stents or tissue scaffolds.

## 1. Introduction

The transformative potential of 3D bioprinting is a cornerstone in tissue engineering and regenerative medicine, enabling the precise fabrication of intricate biological constructs with spatial control over cells and biomaterials [1,2,3]. This technology promises to revolutionise healthcare, from creating patient-specific implants to developing sophisticated in vitro models [4]. The development of functional tissue constructs through 3D bioprinting critically relies on scaffold architecture and the material used, demanding precise mechanical properties and high-resolution fidelity [5]. Photocurable resins—which solidify rapidly under light via techniques like SLA/DLP—are a premier material class due to their ability to fabricate complex, high-resolution objects [6].

However, the successful translation of bioprinted structures into functional tissues hinges critically on overcoming challenges related to bioink formulation, scaffold architecture, vascularisation, and functional tissue maturation [7]. The scaffold architecture design is paramount, as it defines the mechanical environment for cellular activities, nutrient transport, and overall tissue integration. Traditional scaffold designs often suffer from limitations in mimicking the complex anisotropic and heterogeneous mechanical properties of native tissues, which can hinder proper cell differentiation and tissue development [8].

To address these limitations, innovative strategies for scaffold design are urgently needed. Auxetic materials, characterised by their negative Poisson’s ratio—meaning they expand laterally when stretched axially—offer a unique opportunity to engineer scaffolds with superior mechanical properties [9,10]. This counter-intuitive behaviour leads to possible enhancements in various fields of engineering, medicine, sport, and fashion due to their unique deformation behaviours [10,11]. Integrating auxetic geometries into 3D bioprinted scaffolds could provide a mechanical environment that better mimics physiological conditions, promoting more robust tissue regeneration. However, isotropic mechanical properties offer a significant, yet often overlooked, benefit for tissue engineering scaffolds, particularly for load-bearing applications where forces are complex and multi-directional [12,13,14]. An isotropic structure ensures that the mechanical stimulation is uniform regardless of the loading direction, which is ideal for simplifying experimental control and promoting homogeneous tissue formation [15]. However, a significant challenge with many auxetic structures—for example, those based on re-entrant or chiral designs [16,17]—is their inherent anisotropy, meaning their mechanical behaviour (including Poisson’s ratio) varies significantly depending on the loading direction. This directional dependence can introduce non-uniform stresses and strains. Work at the nano level using molecular dynamics studies presents the basic idea of isotropy in 2D auxetic system [18] and, in recent study, also in 3D [19].

The design of the proposed axisymmetric chiral structures (ACSs), especially the appropriately optimised ACS (optACS), aims to mitigate this common drawback by utilising a geometry that, while auxetic, provides an axisymmetric mechanical response. This offers a critical advantage for reliable and reproducible structures. The ACSs are ideal for filling the load-carrying tubes in practical impact-protecting applications or for use as scaffolds or stents in biomedicine applications due to their excellent energy absorption capabilities and unique deformation behaviour [20,21]. The auxetic medical stents, which were developed recently, are based on a geometrically modified missing rib unit cell, where the optimised design demonstrated superior performance by achieving the highest expanded opening percentage while drastically reducing the recoil percentage, thereby enhancing the stent’s effectiveness and minimising potential arterial tissue damage [22].

ACS’s mechanical deformation behaviour and energy absorption capabilities can be further enhanced with the introduction of graded porosity [23]. This can be performed through a simple parametric study, as presented in [23], with discrete concentric regions of different strut thicknesses, or more sophisticatedly by applying structural optimisation, as presented in [24]. However, these structures possess complex 3D geometry, which conventional technologies cannot produce. The emergence of resin additive manufacturing (3D printing) technologies, specifically stereolithography (SLA) and digital light processing (DLP), has further expanded the capabilities for fabricating complex, high-resolution micro-architectures [25,26]. Unlike extrusion-based methods, resin printing allows for the creation of intricate internal geometries with feature sizes often below 100 micrometres, offering unparalleled precision in scaffold design. This high-resolution capability is critical for constructing finely tuned auxetic structures that depend on precise geometric features to exhibit their characteristic mechanical response. The ability to use biocompatible and photo-crosslinkable resins further bridges the gap between advanced manufacturing and biological applications [27].

This paper presents the results of experimental testing of advanced 3D metamaterials, which can also be used as bioprinting scaffolds. The design and fabrication of optACS using resin 3D printing is described in detail, focusing on leveraging topology optimisation to engineer complex chiral unit cells that inherently exhibit auxetic behaviour, while imposing axisymmetric constraints to ensure structural consistency and scalability. The successful fabrication of these intricate structures using photocurable resins demonstrates the feasibility of this principle. Through comprehensive mechanical characterisation, we aim to show that these high-resolution, auxetic structures provide superior mechanical properties. Our findings significantly address the critical need for innovative architectures in 3D bioprinting, paving the way for the development of more functional tissue constructs.

## 2. Materials and Methods

### 2.1. Design

**Figure 1:**
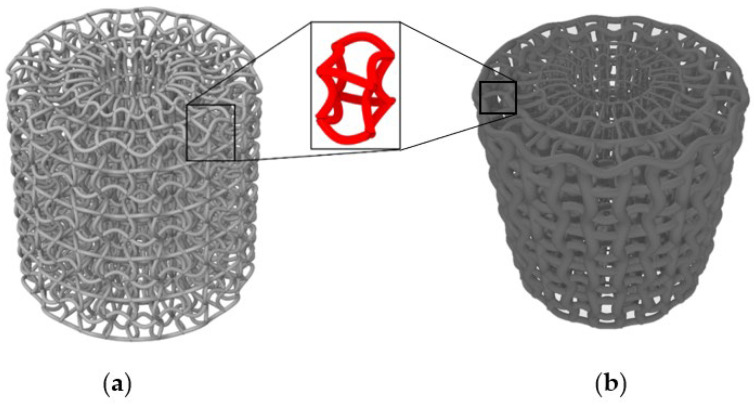
Geometry of uniform ACS (**a**) and optimised optACS (**b**).

The auxetic ACS was developed and patented recently [23,28]. Its architecture is based on chiral unit cells, which feature central nodes interconnected by tangentially attached ligaments, as shown in Figure 1a. The auxetic effect results from the predictable rotation of these nodes under axial strain. The entire lattice is configured with an axisymmetric distribution, meaning the unit cells are periodically mapped around a central axis to form a tubular macrostructure. This axial symmetry and the transformation of the unit cells result in a non-uniform stress field in the case of uniform ACSs [23]. Therefore, computational optimisation was included to achieve a more isotropic stress field in the structure [24], which is specifically employed to mitigate the inherent mechanical anisotropy of many auxetic designs, yielding a structure with a more predictable and near-isotropic response in the plane perpendicular to the loading axis, thus ensuring uniform mechanical properties. The optACS geometry is radially graded with different thicknesses of the struts, as shown in Figure 1b [23].

### 2.2. Fabrication

**Figure 2:**
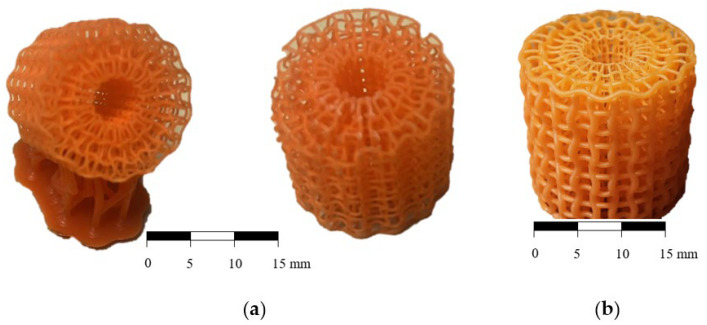
Fabricated uniform ACS (**a**) and optimised optACS (**b**).

High-resolution resin 3D printing was used to fabricate the optimised scaffolds, confirming excellent structural accuracy and fidelity. Specimens were fabricated using a Prusa SL1S 3D resin printer with Prusa brand photopolymer resin (Prusa Research a.s., Prague, Czech Republic). The finest available print settings were selected to accommodate the small dimensions and ensure high surface fidelity. A layer height of 0.025 mm was used, with an exposure time of 1.4 s per layer. Specimens were printed at a 45° angle with supports applied from the bottom to enhance print quality and dimensional stability, as seen in Figure 2.

Printing and post-processing were conducted under ambient laboratory conditions, with temperature maintained at room level and relative humidity within the normal range. Following printing, specimens underwent post-processing using ultraviolet light curing on the Prusa CW1S unit. Washing was performed for 1 min to remove residual uncured resin, followed by a 3 min UV curing cycle to ensure complete polymerisation of the material. During curing, the resin solidifies and cross-links but does not lose mass significantly. Some very minor shrinkage (usually <5%) may occur, which can slightly increase density. The resulting density of the solid material reported in the literature is approximately 0.9975–1.1875 g/cm^3^ [29], which was also used to calculate the relative density of ACS, as shown in Table 1. The relative density of the ACS was calculated by dividing the weight of the fabricated samples by the weight of the solid cylinder of bulk material with same outer dimensions as the samples.

Figure 2 shows the fabricated samples, where the building direction and supports are shown in Figure 2a. Due to optimised geometry, the outer strut thicknesses are increased in the case of optACS (Figure 2b), which results in a higher relative density of optACS samples (Table 1).

### 2.3. Mechanical Testing

The mechanical environment of native tissue must be replicated to successfully apply 3D (bio)printed scaffolds in regenerative medicine. For load-bearing applications, the constructs must withstand complex, dynamic forces. Therefore, at least uniaxial mechanical compression testing is essential for validating novel scaffold designs. Testing optACSs printed via resin 3D printing is crucial for precisely determining their mechanical and deformation behaviour. Specifically, it is necessary to quantify the compressive mechanical behaviour and yield strength to ensure the constructs fall within the physiological range of the target tissue. To accurately measure the full-field strain field and simultaneously axial and lateral strains, it is necessary to employ non-contact methods, such as Digital Image Correlation (DIC). This rigorous approach provides a precise Poisson’s ratio analysis to confirm the structure’s functionality by verifying the hypothesised negative Poisson’s ratio, which is key to the structure’s enhanced resilience and conformability. This comprehensive mechanical assessment also enables comparative evaluation of the uniform and optimised ACS geometries and the resulting physical performance necessary for successful biological integration.

The compressive mechanical properties of the 3D-printed structures were determined by using a uniaxial testing machine (Tinius Olsen TMC, Horsham, PA, USA) at 0.1 mm/s and 8.3 mm/s/cross-head speeds for the quasi-static (QS) and dynamic (DYN) testing, respectively. The recorded load–displacement data were converted to engineering stress–strain values using the initial specimen’s dimensions. The plateau stress was calculated as the average stress in the range of 20–40% compressive strain [30]. Specific Energy Absorption (SEA) values were calculated by integrating compression responses up to a particular strain and dividing by the specimen density. The Crash Force Efficiency (CFE) was calculated as the ratio between the maximum and average stress up to 40% strain. The CFE provides information about the uniformity of the mechanical response, where the ideal crash absorber has CFE = 1.

Image acquisition for Digital Image Correlation was performed using a Canon 70D DSLR camera, which provided sufficient temporal resolution for capturing the deformation process. Digital Image Correlation (DIC) analysis was performed using NCorr, an MATLAB R2022a-based open-source DIC software (Mathworks Inc., Natick, MA, USA) [31].

To precisely quantify the longitudinal and transversal deformation behaviour of tensile specimens and determine the engineering Poisson’s ratio, a Video Image Analysis (VIA) method was used, implemented through custom code built upon the Accord.NET framework, which has also been successfully used in previous research [32]. This technique provides a non-contact means of measuring geometric changes throughout the loading process. The VIA method involved several key automated steps. First, various image filters were applied to the recorded video frames to accurately segment the specimen from the background, thereby defining its edges. The software then automatically determined the time-dependent minimum width of the specimen by calculating the shortest distance between these segmented edges. This continuous measurement provided the crucial transversal strain data required for deformation analysis. Finally, the time-dependent engineering Poisson’s ratio was calculated by taking the ratio of the transversal strain to the longitudinal strain. The time variable, which indexed the captured video frames, was subsequently mapped directly to the engineering strain variable, allowing for a complete characterisation of the specimen’s Poisson’s ratio evolution as a function of its compression strain.

**Figure 3:**
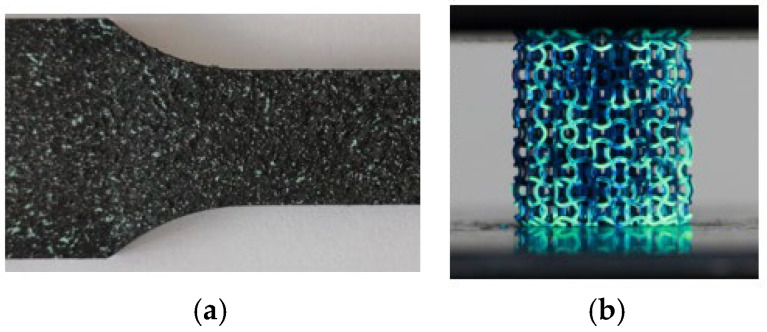
Typical speckled pattern for optical DIC (**a**) and UV pattern (**b**).

In addition to the VIA method, DIC was used to evaluate the true Poisson’s ratio. Typically, DIC requires a high-contrast, random speckle pattern on the surface to facilitate accurate tracking of displacement and strain fields on the structure [33]. This is usually achieved by applying a thin, uniform base coat (commonly white), followed by a random application of a contrasting speckle layer (commonly black) using spray paint or airbrush techniques, as seen in Figure 3a. The quality of the speckle pattern—specifically its contrast, size, and distribution—directly influences the spatial resolution and accuracy of the correlation analysis. Given the small size of the printed specimens, conventional speckling methods were unsuitable. As an alternative, a thin layer of ultraviolet (UV)-reactive paint was applied using a fine spray technique, as seen in Figure 3b. This approach provided sufficient contrast under UV illumination, enabling effective tracking of surface features during DIC analysis. Additionally, although a thin speckled paint layer applied to such small-dimension struts may influence results, UV particles are of much smaller particle size, and their influence on the structure is negligible. The speckle pattern quality was visually inspected to ensure adequate distribution and resolution for the measurement scale.

The true Poisson’s ratio was calculated using displacement fields obtained from Ncorr DIC analysis for comparative purposes. The Ncorr output provided horizontal (u) and vertical (v) displacement data for each image in the deformation sequence. The specimen boundaries were identified for each image using edge detection techniques, and a bounding box was fitted around the detected contour. The midpoint of this bounding box was used to define the specimen’s geometric centre. The top and bottom edges were located along the vertical (v) direction relative to this midpoint, while the left and right edges were identified along the horizontal (u) direction.

Multiple points (typically 50) were sampled along each edge to improve robustness, and the mean displacement values were used for subsequent calculations. Axial deformation was determined from the difference between the mean vertical displacements of the top and bottom edges, while transverse deformation was obtained from the difference between the mean horizontal displacements of the left and right edges. This procedure integrates several numerical methods in sequence—Digital Image Correlation, edge detection, bounding-box geometry, and data averaging. As DIC inherently introduces minor frame-to-frame inaccuracies, averaging multiple edge points helps smooth these fluctuations and yields a more consistent and reliable estimation of Poisson’s ratio with controlled numerical error.

## 3. Results

**Figure 4:**
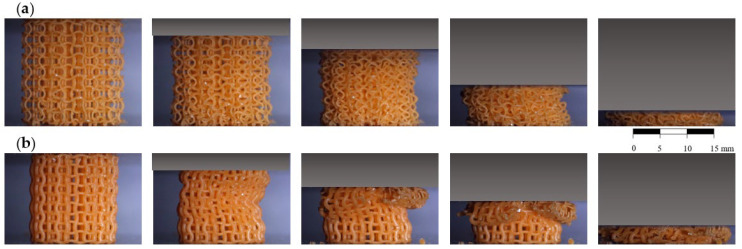
Deformation behaviour of ACS (**a**) and optACS (**b**).

The deformation behaviour under compression loading for ACS and optACS samples is illustrated in Figure 4. Clear auxetic behaviour is visible in both analysed cases; however, in the case of ACS (Figure 4a), the structure buckling is less evident, and therefore the auxetic effect is visually observed until larger strains. This is carefully examined in Section 4.

**Figure 5:**
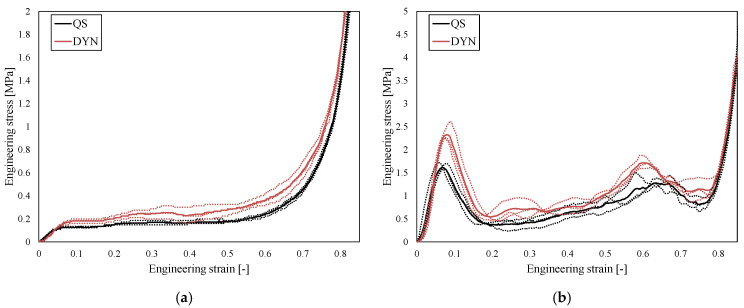
Mechanical responses of ACS (**a**) and optACS (**b**) structure (dashed lines—individual samples, solid lines—average responses).

**Figure 6:**
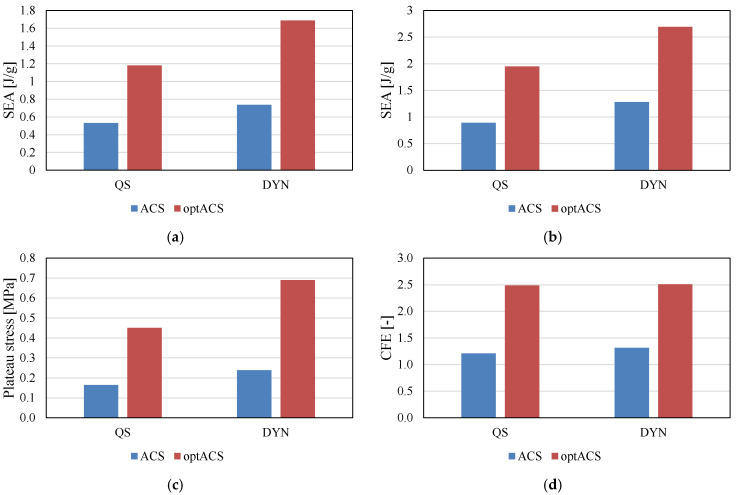
SEA to 40% (**a**), 60% (**b**), plateau stress, (**c**) and CFE (**d**).

The mechanical responses are shown in Figure 5. The individual responses (dotted lines) and the average responses (solid lines) are shown for different geometries and strain rates. As can be observed, a significant strain-rate hardening is present in DYN testing results for both structures, which is a consequence of inertial effects. Due to the higher relative density, the optACS samples achieved higher stiffness, so the direct comparison should be performed with normalised quantities such as SEA, shown in Figure 6.

Figure 6 illustrates differences in SEA at 40% and 60% strain, plateau stress, and the CFE of ACS and optACS at different strain rates. The SEA analysis shows that optACS exhibited superior properties compared to ACS at both 40% (Figure 6a) and 60% (Figure 6b) longitudinal strains. The optACS also exhibit a much higher plateau stress, where it can also be observed that strain-rate hardening is much more pronounced in these structures (Figure 6c). The CFE values (Figure 6d), i.e., the uniformity of the mechanical response, indicates that the ACS respond in a more uniform manner, which is also evident from Figure 5. The drop in stress after reaching the initial yielding is less evident in the case of ACS structure than in optACS.

## 4. Discussion

**Figure 7:**
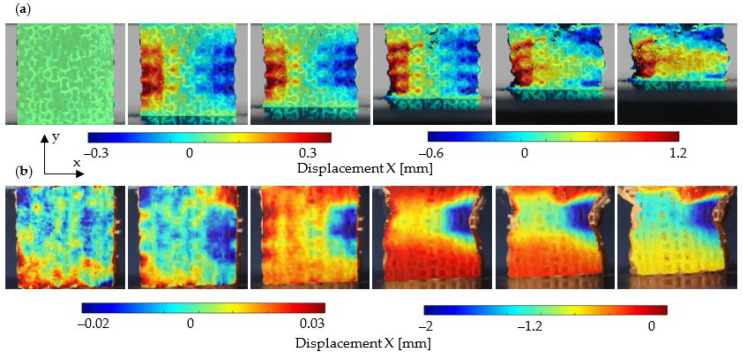
Plot of transverse displacement for ACS (**a**) and optACS (**b**) with displacement increment of 4 mm.

The full-field transverse displacement plot for ACS and optACS is shown in Figure 7, where displacement data are displayed using varying scale factors for improved visual clarity of the displacement field. An apparent auxetic effect is observed for both structures, which is more clearly pronounced in the case of ACS. In contrast, in the case of optACS, local buckling is observed already at the early stages of deformation. There is also an apparent boundary effect in the area of the compression plates, where the structure cannot deform in the designed way due to the friction between the structure and the compression plate.

**Figure 8:**
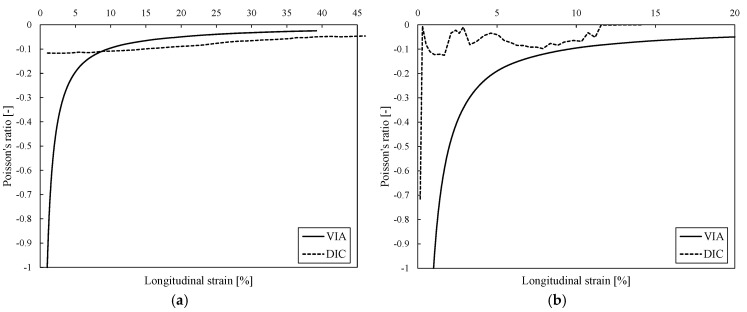
Mechanical responses of ACS (**a**) and optACS (**b**).

Using the DIC and the VIA methods, the engineering and true Poisson’s ratio values and their dependence on the longitudinal strain were calculated (Figure 8). Using both methods, the auxetic effect in the samples was shown, achieving Poisson’s ratio of about −0.1 up to 45% longitudinal strain. While both DIC and VIA capture the same overall trend in the evolution of the Poisson’s ratio throughout the deformation process, a crucial quantitative difference exists: the true Poisson’s ratio values calculated using DIC are consistently higher in the case of ACS and lower in the case of optACS than those obtained via VIA. This disparity likely stems from DIC’s inherent difficulties with highly localised deformation in optACS. Specifically, as localised strain increases, subset tracking in DIC can lead to underestimating the true transverse strain, resulting in lower computed Poisson’s ratio values than the VIA method, which directly measures the critical minimum width change.

Additionally, the average values of the Poisson’s ratio between 10 and 40% longitudinal strain for ACS and between 5% and 20% longitudinal strain for optACSs were calculated and found to be −0.05 and −0.09, respectively.

## 5. Conclusions

Combining auxetic axisymmetric chiral optimisation with high-resolution resin 3D printing offers a robust and reproducible method for addressing critical challenges in scaffold architecture for 3D bioprinting. This technique enables the fabrication of complex, high-performance constructs with tailored mechanical properties, advancing the development of functional tissue constructs for regenerative medicine.

The compressive mechanical behaviour, auxetic properties, and energy absorption capabilities of the standard axisymmetric chiral structure (ACS) and optimised ACS (optACS) structures were comprehensively investigated. Both geometries clearly demonstrated auxetic behaviour under compression up to large longitudinal strains. However, the deformation analysis indicated that the standard ACS exhibited less pronounced buckling, allowing the visual observation of the auxetic effect to persist until larger strains, suggesting a higher mechanism stability during initial deformation than that of optACS. Analysing the mechanical responses confirmed that both structures displayed significant strain-rate hardening under dynamic loading conditions, attributed to a combination of base material effects and inertial forces. While optACS achieved higher stiffness due to its greater relative density, a direct comparison using the Specific Energy Absorption (SEA) provided a normalised measure of performance, which revealed that optACS exhibited superior energy absorption properties, achieving both a much higher plateau stress and significantly greater SEA values. While the optACS geometry demonstrated superior energy absorption, its mechanical response uniformity was considerably worse. The standard ACS provided a more constant and uniform mechanical response across the strain plateau, as evidenced by its lower CFE. The strain-rate hardening effect was considerably more pronounced in the optACS.

In conclusion, a clear performance trade-off exists: optACS is optimised for higher strength and energy absorption, whereas the standard ACS offers greater deformation uniformity and a more robust auxetic response across larger strain ranges before buckling. The observed auxetic effect (negative Poisson’s ratio) in these structures holds significant promise for biomedical applications, as it allows for materials that contract laterally when compressed and expand when stretched, potentially leading to the development of superior stents, tissue scaffolds, or shock-absorbing implants with enhanced conformability and mechanical compatibility with surrounding biological tissues.

## Figures and Tables

**Table 1:** The properties of the samples (Φ is diameter).

Name	Dimensions	Weight [g] (st. dev.)	Relative Density [%]
ACS	Φ20 × 20 mm^2^	0.66 (0.016)	9.67
optACS		1.38 (0.019)	20.15

## Data Availability

The data supporting this study’s findings are available on request from the corresponding author.
